# A Customized Next-Generation Sequencing-Based Panel to Identify Novel Genetic Variants in Dementing Disorders: A Pilot Study

**DOI:** 10.1155/2020/8078103

**Published:** 2020-08-18

**Authors:** Giuseppe Lanza, Francesco Calì, Mirella Vinci, Filomena Irene Ilaria Cosentino, Mariangela Tripodi, Rosario Sebastiano Spada, Mariagiovanna Cantone, Rita Bella, Teresa Mattina, Raffaele Ferri

**Affiliations:** ^1^Department of Surgery and Medical-Surgical Specialties, University of Catania, Catania, Italy; ^2^Oasi Research Institute–IRCCS, Troina, Italy; ^3^Department of Neurology, Sant'Elia Hospital, ASP Caltanissetta, Caltanissetta, Italy; ^4^Department of Medical and Surgical Sciences and Advanced Technologies, University of Catania, Catania, Italy; ^5^Department of Biomedical and Biotechnological Sciences, University of Catania, Catania, Italy

## Abstract

**Purpose:**

The advancements in the next-generation sequencing (NGS) techniques have allowed for rapid, efficient, and cost-time-effective genetic variant detection. However, in both clinical practice and research setting, sequencing is still often limited to the use of gene panels clinically targeted on the genes underlying the disease of interest.

**Methods:**

We performed a neurogenetic study through an *ad hoc* NGS-based custom sequencing gene panel in order to screen 16 genes in 8 patients with different types of degenerative cognitive disorders (Alzheimer's disease, mild cognitive impairment, frontotemporal dementia, and dementia associated with Parkinson's disease). The study protocol was based on previous evidence showing a high sensitivity and specificity of the technique even when the panel is limited to some hotspot exons.

**Results:**

We found variants of the *TREM2* and *APP* genes in three patients; these have been previously identified as pathogenic or likely pathogenic and, therefore, considered “disease causing.” In the remaining subjects, the pathogenicity was evaluated according to the guidelines of the American College of Medical Genetics (ACMG). In one patient, the p.R205W variant in the *CHMP2B* gene was found to be likely pathogenic of the disease. A variant in the *CSF1R* and *SERPINI1* genes found in two patients was classified as benign, whereas the other two (in the *GRN* and *APP* genes) were classified as likely pathogenic according to the ACMG.

**Conclusions:**

Notwithstanding the preliminary value of this study, some rare genetic variants with a probable disease association were detected. Although future application of NGS-based sensors and further replication of these experimental data are needed, this approach seems to offer promising translational perspectives in the diagnosis and management of a wide range of neurodegenerative disorders.

## 1. Introduction

Dementia comprises a group of degenerative disorders leading to a progressive decline in cognitive function and, in some cases, to changes in behavior and motor impairment, ranging from a slowness in some motor activities to an overt parkinsonism. Dementia affects approximately 47.5 million cases worldwide, with 7.7 million new cases reported annually [1]. Similar to other degenerative disorders, no disease-modifying treatment is available for primary dementia and an early diagnosis is one of the best predictors of the disease outcome [2, 3]. In this context, an in-depth understanding of the molecular basis for dementia can provide a path for an early diagnosis and the development of new targeted treatment modalities.

As in any other degenerative disease, genetics is a critical risk factor for dementia, with 5-10% of the cases being familial, often attributed to different genes [4, 5]. However, it is likely that evaluating the incidence of familial cases based only on clinical observation leads to an underestimation of the real number of cases, since other medical conditions may be the cause of death for presymptomatic individuals before the onset of neurodegeneration. Furthermore, genetic testing is still not a universally recommended diagnostic tool for dementia [6–8]. Thus, clinicians who choose to use genetic testing often engage in the screening of a small subset of genes, with a focus on the genotype of patients with known variants with high penetrance, rather than on the sequencing of all genes of the disease. These clinical considerations and the high cost of the testing lead to a significant bias in the estimation of the incidence rates, which cannot be considered epidemiologically accurate.

As a general rule, degenerative cognitive and movement disorders may be caused, at least in part, by single, pathogenic variants (monogenic) or by multiple, small effect, variants (oligogenic) that synergistically act to mediate disease expression [9]. In this context, the application of new methods, such as the next-generation sequencing (NGS) techniques, has led to rapid and efficient genetic variant detection along with a reduction of the cost.

Three types of NGS applications are currently available in the clinical setting: the whole-genome sequencing (WGS), the whole-exome sequencing (WES), and the targeted gene panels [10]. While WGS is a nonspecific approach evaluating the entire genome information of an individual, WES targets only the protein-coding regions of the genome, based on the evidence that disease-associated variants are significantly overrepresented in their coding regions. However, although WES is the most frequently used approach, it has some limitations [10]. For instance, its cost still remains high when employing adequate coverage, which makes the cumulative cost for studies using large sample sizes expensive. A second challenge is that this approach often provides an excessive amount of genetic variations from the exome that often overwhelms researchers, especially if a genetic diagnosis is required for genotype-specific treatments. Another issue is that it generates secondary findings that are sometimes unrelated to the disease of interest [11]. Conversely, the use of targeted gene panels focuses on the specific genes potentially underlying the disease under investigation [9].

The recent improvement of technology employed in the genetic testing of neurodegenerative disorders has seen the introduction of powerful parallel DNA sequencing methods that allow researchers to systematically screen individual genomes for the sequencing of DNA variations at base-pair resolution [12]. The technological improvement has also helped to address the question on missing hereditability and to uncover novel potentially pathogenic genetic variants. Therefore, the targeted sequencing of a clinically significant gene panel may lead to an efficient technique that is also cost-time effective when compared to the use of Sanger sequencing [13].

In this pilot study, we used an *ad hoc* NGS-based custom-designed sequencing gene panel to identify genetic variants in patients with degenerative cognitive disorders, namely, Alzheimer's disease (AD), mild cognitive impairment (MCI), frontotemporal dementia (FTD), and dementia associated with Parkinson's disease (PD). In clinical practice, their identification and differential diagnosis are often challenging, and despite the improvements in diagnosis due to biomarker testing, dementias can have overlapping symptoms and may share common genetic background [14]. In this scenario, genetic testing can help the diagnosis, uncover the specific etiology of the disease, provide information for the family, and indicate eligibility for clinical trials.

The tool used in this study allowed to screen for variants in 16 genes implicated in neurodegenerative disease pathways. The study protocol was based on previous evidence by Beck and colleagues [13], who showed a high sensitivity and specificity of the technique even when the panel is limited to some hotspot exons. More work is needed to improve information available in the literature and databases about the pathogenicity and penetrance of variants. For this reason, the further molecular characterization of patients helps to provide additional evidence for the clinical and mutational heterogeneity of dementia and to better define the genotype-phenotype correlation. This is relevant not only for a deeper understanding of dementing processes but also for the genetic counseling and therapeutic approach of patients and relatives.

## 2. Materials and Methods

### 2.1. Participants

The genetic panel was tested in 8 patients (4 females) with one of the following clinical diagnosis: AD (*n* = 2), MCI (*n* = 2), FTD (*n* = 2), and PD-associated dementia (*n* = 2). The age at the time of examination ranged from 34 to 87 years, whereas the age range at onset was between 32 and 84 years.  Table 1 summarizes the relevant clinical and demographic data and the main laboratory and instrumental findings. All participants were Caucasian and of Sicilian ancestry.

Family history was collected through a detailed interview with a first-degree relative or the proband spouse. The clinical and past medical history of each patient was collected, and all of the available documents related to the affected members (e.g., medical records, certificates, and drug prescriptions) were acquired. In four subjects (patients 2, 4, 5, and 6), a family history of neurodegenerative disease was reported, whereas the other four cases were considered sporadic, as determined by the patient recall and confirmed by the caregivers. All clinical diagnoses were supplied by a trained neurologist, in accordance with the current diagnostic criteria for AD [15], MCI [16], FTD [17], and PD-dementia [18]. Recruitment occurred between July 2017 and October 2018.

All subjects (or their legal guardians) gave their informed consent for inclusion before they participated in the study. The study was conducted in accordance with the Declaration of Helsinki of 1964 and its later amendments, and the protocol was approved by the Ethics Committee of the Oasi Research Institute–IRCCS in Troina, Italy (approval code: 2018/07/18/CE-IRCCS-OASI/14).

### 2.2. NGS Sequencing

Blood samples were collected from all patients. DNA and plasma were obtained according to standard procedures. Genomic DNA was isolated from lymphocytes using the salt chloroform extraction method, checked for degradation on agarose gel, and quantified by the Qubit 2.0 Fluorometer. The Ion AmpliSeq™ Dementia Research Gene Panel [13] was used to identify genetic variations associated with dementia. This panel contains 214 amplicons in 2 pools.

A polymerase chain reaction amplicon-based library preparation (AmpliSeq Designer software, Life Technologies, CA, USA) was used to screen the following dementia disease genes: *PRNP* (prion protein; Ex2), *APP* (amyloid precursor protein; Ex1, 3, 4, 9, 10, 12, 13, 15-18), *PSEN1* (presenilin 1; Ex2-12), *PSEN2* (presenilin 2; Ex5-8, 13), *GRN* (granulin; Ex1-13), *MAPT* (microtubule-associated protein tau; Ex2, 6-14, coverage 98%), *TREM2* (triggering receptor expressed on myeloid cells 2; Ex1-5), *CHMP2B* (charged multivesicular body protein 2b; Ex5-6), *CSF1R* (colony stimulating factor 1 receptor; Ex12-22), *FUS* (fused in sarcoma; Ex3, 5, 6, 12-15), *ITM2B* (integral membrane protein 2B; Ex6, *coverage* = 98%), *NOTCH3* (notch receptor 3; Ex3-4), *SERPINI1* (serpin family I member 1; Ex2-9), *TARDBP* (TAR DNA binding protein; Ex2-6), *TYROBP* (TYRO protein tyrosine kinase binding protein; Ex1-5), *VCP* (valosin-containing protein; Ex1-17), and *SQSTM1* (sequestosome 1; Ex1, 2-8, *coverage* = 98%), according to Beck and colleagues [13].

Template preparation, clonal amplification, recovery, and enrichment of template-positive Ion Sphere™ Particles and loading of sequencing-ready Ion Torrent semiconductor chips (Ion 314) were performed with the Ion Chef™ System. Sequencing runs were performed using the Ion S5 Sequencing kit (Thermo Fisher Scientific). Data of runs were processed using the Ion Torrent Suite 5.10, Variant Caller 5.10, Coverage Analysis 5.10 (Thermo Fisher Scientific), Ion Reporter (Thermo Fisher Scientific), and/or wANNOVAR tools [19]. DNA sequences were displayed by using Integrated Genomics Viewer [20]. Sanger sequencing was performed to confirm all mutations.

Variants were assessed using the PolyPhen-2, SIFT, MutationTaster, FATHMM, and PROVEAN software tools. Additionally, the CADD database was used to classify variants as harmful or not based on a numerical cut-off value (>20 = *harmful*). We removed all the common variants (*minor* *allele* *frequency* > 1%) reported in the public databases 1000 Genome Project and Exome Sequencing. According to these databases, variants can be classified as tolerated, deleterious, benign, neutral, harmful note, and harmful. Finally, based on the American College of Medical Genetics (ACMG) guidelines [21], an evidence of pathogenicity was assigned to each variant identified as follows: 1 (benign), 2 (likely benign), 3 (uncertain significance), 4 (likely pathogenic), and 5 (pathogenic).

## 3. Results

NGS analysis by using the 16 abovementioned genes panel (*PRNP*, *PSEN1*, *PSEN2*, *APP*, *GRN*, *MAPT*, *TREM2*, *CHMP2B*, *CSF1R*, *FUS*, *ITM2B*, *NOTCH3*, *SERPINI1*, *TARDBP*, *TYROBP*, and *VCP*) was successfully completed in all patients. By using the Ion AmpliSeq™ Dementia Research Gene Panel [13], we saw an average of 98% of bases at >20x coverage. The average read depth per sample was 420x. The number of variants/patient was ~180.

Table 2 illustrates the patients in whom the variants were revealed, as well as the position, gene inheritance pattern, type (splicing, missense, and others), genotype, and which ones were already known. In particular, the variants c.482+2T>C in the *TREM2* gene [22], the c.C613T in the *CHMP2B* gene [23, 24], and the c.G2137A in the *APP* gene [25] were already known. Conversely, the variants c.G2239A in the *CSF1R* gene, the c.G289A in the *SERPIN1* gene, the c.C110G in the *GRN* gene, and the c.G1604A in the *APP* gene were not previously described.

Table 3 shows the results of the *in silico* analysis of the variants that resulted pathogenic according to the established classification criteria and provided data on the allelic frequency in the general population. All Sanger-sequenced variants were in accordance with the NGS results.

## 4. Discussion

### 4.1. Main Findings

The development of NGS sequencing technology has allowed for a rapid and efficient analysis of several genes simultaneously, thus providing significant clinical advantages, especially for the diagnosis of complex diseases with high genetic heterogeneity (i.e., different genes responsible for the same clinical phenotype), such as cognitive impairment and movement disorders.

In this study, we support previous evidence by Beck and colleagues [13] by showing that, although limited to some hotspot exons, the panel has a high sensitivity and specificity. The possibility to screen the main genes involved in dementia, including the early-onset forms, with a good probability of success and at a relatively reduced cost, is the main advantage. On the other hand, the fact that only a few exons in a limited number of genes can be examined is a limitation. The exoma-trios approach, although more expensive, would probably disclose additional results. Nevertheless, prior to ordering genetic testing, clinicians must determine the appropriate genes to test and the best type of genetic test to use. Without this analysis, interpretation of genetic results is difficult. Patients and relatives should be counseled about the benefits and limitations of the different types of genetic tests, so they can make an informed decision about testing.

In our study, the application of a customized panel of 16 dementia-associated genes in 8 patients allowed to identify one or more variants and related pathogenetic role. Some of these variants were not reported before, whereas others were already known. Namely, mutations of the *TREM2* (patients 1 and 2), *CHMP2B* (patient 3), and *APP* (patient 5) genes have already been found to be pathogenic or likely pathogenic in the literature and, therefore, they can be considered “disease causing” [26]. In patient 3, a segregation within the family was not reported, and the same variant has been described also in patients with different phenotypes [23, 24] and not in the general population; therefore, further functional studies are needed.

In the remaining patients, given that the variants were not present in the databases (HGMD, ClinVar), the pathogenicity was evaluated according to the ACMG guidelines [21]. The variants found in patients 4 and 6 (*CSF1R* and *SERPINI1* genes) should be classified as classes 2 and 1, respectively, whereas the other two variants (*GRN* gene in patient 7 and *APP* gene in patient 8) should be both classified as class 4. Of note, the missense p.R535H mutation in patient 8 was located in a relevant functional “collagen-binding” domain of the APP protein. In particular, the specific binding of the APP to extracellular matrix molecules suggests that APP regulates cell interactions and has a function as a cell adhesion molecule and/or substrate adhesion molecule [27].

To summarize, four new variants have been identified in our study. While in patients 4 and 6 the variant has been classified as benign or likely benign, thus not playing a significant pathogenic role, in patients 7 and 8, the *in silico* analysis, the data from CADD_phred, and the absence of the variant in the general population allow to hypothesize a pathogenic role. However, additional functional studies and further data supporting a potential pathogenicity (e.g., allelic frequency in the ethnic population, segregation within the family, effects at the protein level, and protein domain) are needed.

To date, the role for novel variants of unknown significance in both common and rare dementia-associated genes has not been exhaustively elucidated, although some novel, likely pathogenic variants have been recently reported in Italian patients with dementia [28]. In our study, the splicing variant c.482+2T>C was found on the *TREM2* gene in patients 1 and 2, who were not relatives, not even distant. Given the prevalence of this variant in Italy and particularly in the Sicilian population (6/20 alleles, 30%) [22, 29], the occurrence of a “founder effect” might be hypothesized. Further studies with larger samples are necessary, although these data may help in disentangling the role of the genetic variant observed [30].

The present results also support the hypothesis that early-onset dementia may be the result of interconnected mechanisms that lead to neurodegeneration where the implication of the same genes can be seen in one or more systems [31, 32]. Most of the genes tested here play a pivotal role in multiple cellular pathways rather than being involved in a single form of dementia. Some of these pathways include the energy metabolism, the phospholipid and cholesterol efflux, the intracellular and vesicle trafficking, and the neuronal-viability and survival that are usually compromised in every neurodegenerative process and involve several other molecular actors [32].

This implicates a potential additive/synergic effect in early-onset forms of dementia associated with the inter- and intrafamilial expressivity, as recently demonstrated by using a NGS-based analysis in patients with early-onset dementia [12]. For instance, the *TREM2* gene, coding for a microglial lipid sensor that interacts with several factors involved in the metabolism of lipids, could decrease the occurrence threshold of dementia [33]. This means that *TREM2* mutation can cause an aberrant innate immune cell signaling that contributes to several neurodegenerative pathway initiations and propagations [12], including those involved in FTD and PD-dementia [32].

It also appears that early-onset cases are associated with rare variants or risk alleles, which can help in correlating genotype and phenotype. Overall, these findings strengthen the use of an exome/whole NGS approach and stimulate studies on larger samples and with expanded panels of candidate genes. In the specific case of the AD patient carrying the *GRN* mutation, since missense mutations do not affect the progranulin levels, a pathogenic role seems unlikely. However, as no functional study has been performed, we cannot exclude a pathogenic role other than “loss of function”, being this gene implicated in the pathophysiological mechanisms leading to AD [12].

The NGS-based custom-designed resequencing panel used in our study has proven to be a rapid and accurate diagnostic sensor for the in-parallel screening of several neurodegenerative genes, thus allowing to identify disease-specific risk markers and potentially overlapping pathways across the most common dementing diseases. Moreover, after the library preparation, we could analyze the genetic data for 24 samples in less than 30 h.

Finally, data from targeted NGS panels may provide further insights on the genes implicated in neuronal plasticity and microglial neurogenesis. In particular, an intriguing relationship between some causative gene expressions and changes in synaptic morphology and neuronal plasticity has recently been identified. For instance, the *APP* gene family and its products are able to modulate phenomena of hippocampal long-term plasticity [34], as well as the microglial *TREM2* gene might have a role in the synaptic loss depending on the AD stage [35], whereas the *CHMP2* gene regulates synaptic plasticity in dendritic spines [36]. Accordingly, genetic studies could pave the way for the *in vivo* and “real-time” functional evaluation of cortical circuits by using noninvasive brain stimulation techniques (NIBS) [37–39] and even for the NIBS-related neurobiological after-effects (gene activation/regulation, *de novo* protein expression, morphological changes, changes in intrinsic firing properties and modified network activities resulting from changed inhibition, homeostatic processes, and glial function) [40–42]. In patients with causative mutations, genetic findings, coupled with clinical, psychocognitive, neuroradiological, and electrophysiological data, will allow to adopt preventive strategies in the presymptomatic stage, to start treatment since the very early stages of the disease [43–46] and to multidimensionally monitor the disease progression [47, 48].

### 4.2. Limitations

The main limitation of this study is its small sample size that precludes data generalization and further conclusions and, therefore, it should be considered preliminary.

Despite its efficiency and rapidity, NGS approaches have some limitations. One of these is that the sensor cannot discover novel disease loci, since it only captures variants within the selected genes and related exons. Another limitation is that it cannot capture multinucleotide repeat expansions in genes, which is, however, a limitation that characterizes all NGS platforms [49]. Indeed, the current NGS methods cannot help in the detection of some neurological conditions, such as Huntington's disease, Friedreich's ataxia, Fragile X syndrome, myotonic dystrophy, and a subset of spinocerebellar ataxias (diagnoses not included in the present study), that are caused by multinucleotide repeat expansions [50, 51]. Further studies that aim at the identification of new mutations in genes apart from the ones that the exons describe or those located in the conventional splicing sites are needed.

A third limitation lies in the fact that genetic penetrance and expressivity differ because of the modifier genes, allelic variations, environmental factors, and complex environmental and genetic interactions, thus explaining, at least in part, the phenotype differences observed in these patients. Another caveat is that *in silico* analyses require cautious handling and further evidence within the clinical and diagnostic setting to predict the effects produced by each variant needed to refuse or support the pathogenetic role of new variants in the daily clinical practice [30].

Finally, as commonly observed in this type of study, the pathogenicity of genetic variants in late-onset diseases through mechanisms of segregation of the variant within the family is complex and often challenging for different reasons (e.g., the unavailability of DNA from the parents of the patient or a late onset of the clinical phenotype in other family members, such as siblings or cousins). In the present study, the sequencing was not performed in the family members of the four patients with family history of dementia and, therefore, we could not verify whether the affected relatives were carriers of the same variants. The verification of the role played by polygenic risk variants in dementia requires the implementation of a systematic screening of both rare and common variants in several dementia-associated genes and in prospective cohorts.

Notwithstanding the abovementioned limitations and the complex nature of neurodegenerative process and progression, we were able to detect some rare variants with a probable, but not absolutely certain, disease association, based on allele frequency in the general population and the predictive score of multiple *in silico* software. As the etiology of degenerative diseases is often heterogeneous and multiple factors (e.g., dietary intake, traumatic brain injury, vascular disease, infections, or toxin exposure) can confer a variable risk to the disease onset and course, these genetic variants (especially the novel variants) need future validations to determine their effect size and the contribution to disease. Of particular interest are variants in genes with multiple disease associations, as they may provide clues on the development of innovative therapies. This further confirms the evidence that dementia-associated genes do have pleiotropic effects on different neurodegenerative disorders.

## 5. Conclusions

Notwithstanding the preliminary value and the limitations of the study, the “targeted gene” panel used here might allow to increase the number of potentially dementia-related variants and to extend the clinical features associated with genetic variants described in the *TREM2*, *CHMP2B*, *APP*, and *GRN* genes. The development and continuous advances of NGS technologies have opened an exciting window on the molecular diagnostics of several diseases caused by mutations of a large number of genes. Translationally, this technique has demonstrated reliability and accuracy, along with a significant reduction in DNA sequencing costs compared to tests based on the Sanger method. The future application of NGS-based sensors and the further replication of these experimental data will replace the so-called “gene-by-gene” approach with a “panel of genes” strategy that offers promising perspectives in the diagnosis and management of dementia and other neurodegenerative disorders.

## Figures and Tables

**Table 1:** Patients' clinical-demographic data and main laboratory-instrumental findings.

Patient's number	1	2	3	4	5	6	7	8
Sex	M	M	F	F	F	M	F	M
Age	35	34	69	59	71	66	87	85
Parents' consanguinity	—	+	+	—	—	—	—	—
Family history	—	+ (brother)	—	+ (mother, brother)	+ (father, sister)	+ (not specified)	—	—
Age at onset	34	32	66	54	66	65	82	84
Past medical history	Unremarkable	Traumatic brain injury at 1-year old; smoking and cannabis abuse	Hypothyroidism dyslipidemia; lumbar disc protrusions and spondylosis	Mild hypothyroidism	Hypertension; dyslipidemia	Peripheral L facial nerve palsy; R-side sphenoidal meningioma	Diabetes; chronic ischemic heart disease	Duodenal ulcer; benign prostatic hyperplasia
Clinical presentation	Urge incontinence; behavioral changes (irritability, apathy); gait and speech slowness	Behavioral changes (verbal aggressivity, personal carelessness); speech and memory deficit; disorientation; postural instability with some falls	Motor slowness; L hand tremor; progressive memory deficit; depressed mood; episodes of falls; insomnia with excessive daytime sleepiness	Behavioral changes; obsessive thoughts; delirium and complex visual hallucinations (> mysticism); dysphagia; episodes of loss of consciousness; incontinence	Progressive memory deficit and disorientation; slight behavioral changes (apathy, irritability); lack of insight	Progressive speech disorder, with anomia and deficit of object naming; irritability	Progressive memory deficit; loss of personal independence	Progressive memory deficit and disorientation; loss of personal independence; episodes of falls without loss of consciousness; mild bilateral kinetic tremor of the hands
Clinical signs	Hypomimic face; gait and speech slowness; R>L hand postural tremor; L>R upper limb bradykinesia and plastic hypertonus; diffuse brisk tendon reflexes; bilateral Babinski sign; frontal release signs	Gait disorder; mild cerebellar signs; L-beating nystagmus; bilateral palmomental reflex	Hypomimic face; bradykinesia; parkinsonian gait; head and voice tremor; postural instability; L>R upper limb postural and kinetic tremor; L Hoffman sign; bilateral palmomental reflex	Hypomimic face, drooling; akathisia; dysarthria; dysphagia; mandibular contracture; diffuse plastic hypertonus and bradykinesia; bilateral palmomental reflex	Diffuse brisk tendon reflexes; L Hoffman sign; bilateral palmomental reflex	L facial nerve palsy; bilateral sensory-neural hearing loss; anomia, semantic paraphasia	Frontal release signs; diffuse hypoexcitable tendon reflexes	Limping gait; inconstant R hand tremor; diffuse brisk tendon reflexes; positive frontal release signs
ADL IADL	6/6 8/8	4/6 5/8	5/6 3/8	2/6 0/8	6/6 8/8	6/6 8/8	4/6 0/8	3/6 0/8
Neuropsychologic evaluation	Moderate major neurocognitive disorder	Severe major neurocognitive disorder with behavioral changes	Mild neurocognitive disorder	Severe major neurocognitive disorder with behavioral changes	Mild neurocognitive disorder	Mild neurocognitive disorder	Severe major neurocognitive disorder	Severe major neurocognitive disorder
Extensive laboratory exams	Normal	Folate: 7.0 nmol/l (n.v. 10.4-42.4); homocysteine: 37.6 *μ*mol/l (n.v. 3.6-15.0)	Thyroid stimulating hormone: 6.8 mcrUI/ml (n.v. 0.3-4.2)	Erythrocyte sedimentation rate: 50 mm/h (n.v. 2-12)	Low-density lipoprotein: 165 mg/dl (n.v. 0-100)	Prostate specific antigen: 9.2 ng/ml (n.v. 1.0-5.4)	Glycated hemoglobin: 6.1% (n.v.<6.0)	Hemoglobin 10.7 g/dl (n.v. 13.0-17.5); free thyroxine 28.1 pg/ml (n.v. 9.3-17.0)
EEG	Normal	Low-amplitude alpha rhythm; sporadic muscular activations, not correlated with EEG changes	Normal	Normal	Normal	Sporadic slow activity over the frontal and temporal regions	Diffuse slow activity	Diffuse slow activity
Brain MRI	Diffuse cortical and subcortical atrophy (>midbrain, and corpus callosum); ischemic WMLs (> periventricular and frontal regions)	Diffuse cortical and subcortical atrophy (>frontal and temporal lobes, corpus callosum); multiple ischemic WMLs (> periventricular)	Diffuse cortical atrophy; chronic vascular lesion of periventricular frontal regions; mild ischemic WMLs	Diffuse cortical and subcortical atrophy (>frontal and perisylvian regions); mild ischemic WMLs	Moderate cortical atrophy (>frontal and temporal regions); mild ischemic WMLs	R-side parasellar meningioma; moderate diffuse cortical atrophy; mild ischemic WMLs	Diffuse cortical and subcortical atrophy	Diffuse cortical and subcortical atrophy
Other exams	EMG: normal. Multimodal EPs: normal. CSF: total tau: 266 pg/ml (n.v. 136 ± 89). Perfusional SPECT: bilateral frontal, parietal, and temporal hypoperfusion. DAT-scan SPECT: L>R nigrostriatal denervation	EMG: normal. Multimodal EPs: normal. CSF analysis: normal	Unremarkable	EMG: diffuse neurogenic changes (>bilateral deltoid and right biceps brachii muscles); no cranial muscle denervation. Spine MRI: disc protrusion C5-C6, L4-L5, and L5-S1; spondylosis	Transthoracic echocardiogram: L ventricle enlargement	Supra-aortic vessels ultrasound: bilateral carotid artery thickening	Chest X-ray: signs of chronic obstructive pulmonary disease. Transthoracic echocardiogram: L ventricle hypertrophy and moderate mitral valve insufficiency	Chest X-ray: signs of chronic obstructive pulmonary disease. Transthoracic echocardiogram: L ventricle enlargement
Diagnosis at discharge	PD-dementia	FTD	PD-dementia	FTD	MCI	MCI	AD	AD

Legend (in alphabetic order): -=absent/negative; +=present/positive; AD=Alzheimer's disease; ADL=activity of daily living; CSF=cerebrospinal fluid; DAT=dopamine transporter; EEG=electroencephalogram; EPs=evoked potentials; FTD=frontotemporal dementia; F=female; IADL=instrumental activity of daily living; L=left; M=male; MCI=mild cognitive impairment; MRI=magnetic resonance imaging; n.v.=normal values; PD=Parkinson's disease; R=right; SPECT=single-photon emission computed tomography; WMLs=white matter lesions.

**Table 2:** Patients' genetic features.

Patient's number	Chromosome	Gene	Inheritance pattern	Mutation type	Variant	Protein	Genotype	Reference	dbSNP number
1	6	*TREM2*	Autosomal recessive	Splicing	c.482+2T>C	—	Homozygous	Paloneva et al. [22]	rs386834144
2	6	*TREM2*	Autosomal recessive	Splicing	c.482+2T>C	—	Homozygous	Paloneva et al. [22]	rs386834144
3	3	*CHMP2B*	Autosomal dominant	Missense	c.C613T	p.R205W	Heterozygous	Kim et al. [23] Zhang et al. [24]	rs373536428
4	5	*CSF1R*	Autosomal dominant	Missense	c.G2239A	p.G747R	Heterozygous	This study	rs41355444
5	21	*APP*	Autosomal dominant	Missense	c.G2137A	p.A713T	Heterozygous	Carter et al. [25]	rs63750066
6	3	*SERPINI1*	Autosomal dominant	Missense	c.G289A	p.V97I	Heterozygous	This study	rs61750375
7	17	*GRN*	Autosomal dominant	Missense	c.C110G	p.A37G	Heterozygous	This study	No data
8	6	*APP*	Autosomal dominant	Missense	c.G1604A	p.R535H	Heterozygous	This study	No data

**Table 3:** Results of the *in silico* analysis, along with evidence of pathogenicity.

Patient's number	Gene	Variant	SIFT	PolyPhen-2 HDIV	MutationTaster	FATHMM	PROVEAN	CADD_phred	1000 Genomes (allele frequency)	Pathogenicity (ACMG guidelines) [21]	ClinVar
1	*TREM2*	c.482+2T>C							No data	5	Likely pathogenic
2	*TREM2*	c.482+2T>C							No data	5	Likely pathogenic
3	*CHMP2B*	c.C613T							No data	4	No data
4	*CSF1R*	c.G2239A	T	B	N	D	N	8.944	0.001	2	Likely benign
5	*APP*	c.G2137A							No data	5	Likely pathogenic
6	*SERPINI1*	c.G289A	T	B	N	D	N	0.024	0.002	1	Benign/likely benign
7	*GRN*	c.C110G	T	D	N	T	N	20.4	No data	4	No data
8	*APP*	c.G1604A	T	D	D	T	N	28.5	No data	4	No data

Legend: A=harmful note; B=benign; D=deleterious; N=neutral; T=tolerated; ACMG=American College of Medical Genetics; 1=benign; 2=likely benign; 3=uncertain significance; 4=likely pathogenic; 5=pathogenic.

## Data Availability

The authors declare that all the data regarding this submission are fully available within the manuscript.
